# Coronary artery bypass surgery in diabetic patients – risk factors for sternal wound infections

**DOI:** 10.3205/iprs000097

**Published:** 2016-07-28

**Authors:** Kristina Lenz, Michael Brandt, Sandra Fraund-Cremer, Jochen Cremer

**Affiliations:** 1 Department of Cardiac and Vascular Surgery, University Hospital Schleswig-Holstein, Campus Kiel, Germany

**Keywords:** coronary artery bypass surgery, CABG, diabetes mellitus, sternal wound infection, sternal osteomyelitis

## Abstract

The incidence of sternal wound infections (SWI) after coronary artery bypass surgery (CABG) as reported worldwide is low. However, it is associated with significant increase of postoperative mortality and treatment costs. The major risk factors discussed are diabetes mellitus and bilateral IMA harvesting of the internal mammary artery. This study analyses data of 590 patients receiving CABG concerning the risk factors for SWI. Sternal wound infections occur significantly more often in diabetic patients, one crucial and significant additional risk factor is obesity.

## Zusammenfassung

Die Inzidenz sternaler Wundinfektionen (SWI) nach aorto-koronarer Bypasschirurgie (CABG) ist weltweit als niedrig beschrieben. Als wesentliche Risikofaktoren werden Diabetes mellitus und bilaterale Entnahme der A. mammaria interna diskutiert. Die vorliegende Studie analysiert die Daten von 590 Patienten nach aorto-koronarer Bypasschirurgie hinsichtlich des Risikos sternaler Wundinfektionen. Sternale Wundinfektionen treten bei Diabetikern signifikant häufiger auf. Ein zusätzlicher wesentlicher und signifikanter Risikofaktor stellt erhöhtes Körpergewicht dar.

## Introduction

Although the incidence of severe infections following medial sternotomy is low as worldwide reported: 0.43 to 2.3% [1], the effects on the periprocedural outcome are enormous. The overall mortality is rising from 1.6 to 7.3% [2], the length of in-hospital-stay and the costs of the treatment show high economic relevance. 

Patients with the need of coronary artery bypass surgery are often diabetic.

A wide range of definitions and reported incidence of the complicated sternal wound makes a comparison of the data difficult. 

The guidelines of the Center for Disease Control and Prevention discriminate the superficial sternal wound infection (SSWI) from the deep sternal wound infection (DSWI). The SSWI affects the skin and subcutaneous tissue of the incision site, whereas the DSWI is defined as an infection of the tissue beneath the subcutaneous layer.

Additional aspects of DSWI are:

1. Organism isolated from culture of the mediastinum
2. Clinical findings for mediastinitis
3. Chest pain, instability of the sternum, fever >38°C.

The classification of El Oakley and Wright is based on the above guidelines but includes also delay of superficial wound healing without severe consequences that require the further surgical treatment [1]. According to this classification there is an incidence of 2.6 to 10.1% of SWI after median sternotomy. 

The risk factors for SWI can be devided in technical aspects of the operation such as edema, necrosis, contamination, ischaemia, and adaption of the tissue. The majority of risk factors is patient specific such as metabolic status, immune-competence or malnutrition. There are lots of data without significant outcome. The most relevant factors are obesity (BMI >30kg/m^2^), chronic obstructive pulmonary disease (COPD), diabetes mellitus, and the use of the internal mammary artery (IMA), especially bilaterally.

This study evaluates the risk factors and outcome after CABG in diabetic patients.

## Methods

This is a retrospective analysis of the data of 675 patients of the Clinic of Cardio-Vascular Surgery of the University of Schleswig-Holstein, Campus Kiel. Because of incomplete data or combination of different surgical techniques there were some exclusions. In summary there were 590 patients included in the analysis. 

All patients received a median sternotomy for CABG. Saphenous vein and IMA served as arterial grafts. The operations were performed as off-pump with mild hypothermia. All patients were treated postoperatively at the intensive care unit (ICU). In case of hyperglycemia >120mg/dl blood sugar control was achieved by continuous i.v. insulin application.

Wound infections were diagnosed clinically and divided in three groups:

1. Superficial without further treatment (Grade A)
2. Deep incisional infection with affliction to the periostal tissue layer (Grade B)
3. Sternal surgical revision required (Grade C)
4. Sternal instability (Grade D).

For statistical analysis we did not anticipate normally distributed data, and we applied for multiple comparison the Kruskal-Wallis rank sum test. SPSS for Windows software was used. Significance level was set for p≤0.05.

## Results

137 of the 590 patients were diabetic (23%), 39% of them suffered from insulin-dependent diabetes mellitus (IDDM). 

**Figure 1:**
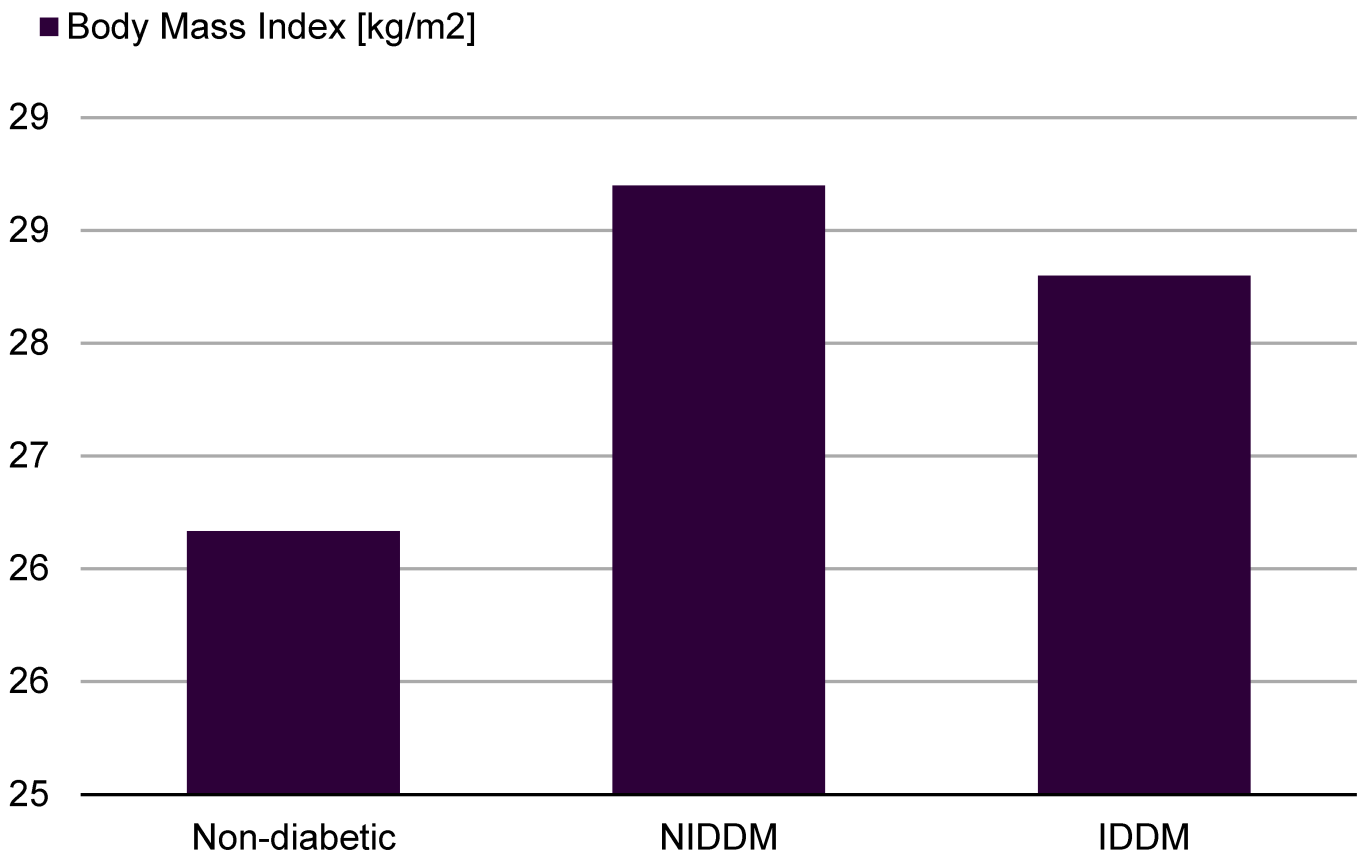
Relation of body mass index. NIDDM: non-insulin-dependent diabetes mellitus, IDDM: insulin-dependent diabetes mellitus.

The body mass index (BMI) was significantly higher in the diabetic group than in the non-diabetic group, serving as control group: 28.75 vs 26.53 kg/m^2^ (p<0.001) (Figure 1 (Fig. 1)). 

The duration of operation and the number of grafts were similar in the groups, also the use of single (IMA) and bilateral (BIMA) internal mammary artery grafts.

The patients stayed 3 to 4 days at the ICU after operation (medians: non-diabetic 2.8 days, NIDDM 3.0 days, IDDM 3.0 days; non-significant) and demission mostly to a rehabilitation institute was realized after 11 days (medians: non-diabetic 10.8 days, NIDDM 10.7 days, IDDM 10.7 days; non-significant). These parameters did not differ between the groups, neither the period of mechanical ventilation.

**Figure 2:**
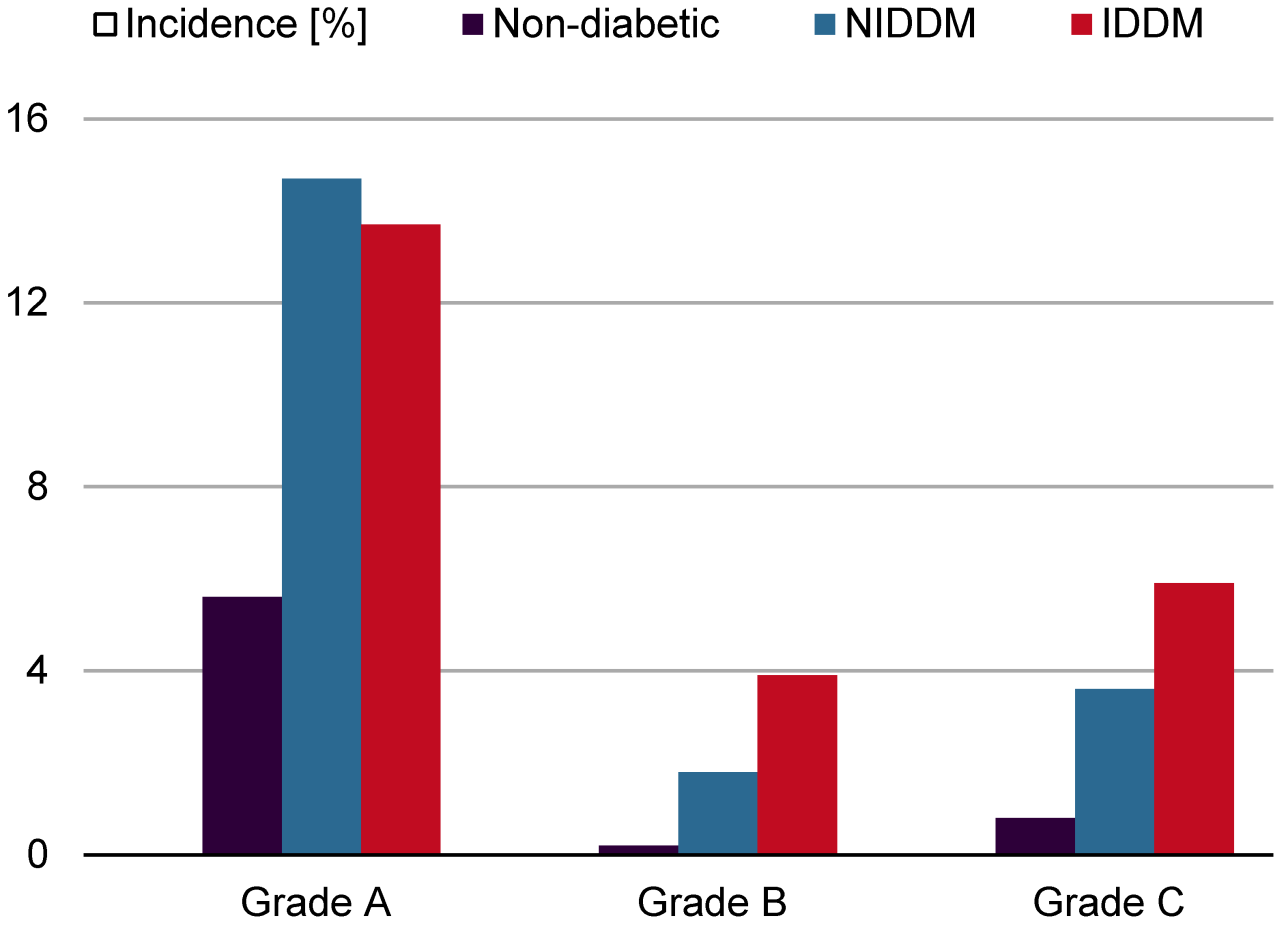
Incidence [%] of different types of superficial wound infections (SWI). NIDDM: non-insulin-dependent diabetes mellitus, IDDM: insulin-dependent diabetes mellitus.

Concerning the healing process of the sternotomy there were significant differences between the three groups. Especially in the IDDM group there was a significant higher rate of superficial sternal wound infections (SSWI) and deep sternal wound infections (DSWI) (Figure 2 (Fig. 2), Table 1 (Tab. 1)).

Infections of the saphenous graft site occured more often but without significant differences between the groups as well as the incidence of the less frequent complication of unstable sternum. 

There was no significant difference concerning the in-hospital-mortality (medians: non-diabetic 3.3%, NIDDM 2.4%, IDDM 3.7; p=0.739).

## Discussion

The high prevalence of SWI in the diabetic groups in this study is concordant to previously published studies [3], [4]. This is related to obesity as additional risk factor.

Further preoperative factors such as obstructive pulmonary disease or chronic kidney failure did not show higher incidence in the diabetic groups.

A current discussion is still concerning the use of IMA/BIMA for grafting. The arterial blood supply of the human sternum is considered to be derived from the arterial periostal plexus which is fed by segmental sternal branches of the IMA [5]. There are three types of collateral vessels, two of them originate also from the IMA, the third is a persistent posterior intercostal artery. 

Former studies found an increase of the odds ratio of 13.9 in diabetic patients caused by bilateral use of IMA grafts [6], [7]. Because of the superior long-term outcome especially of diabetic patients with a reduction of the cardiac 5-year-mortality from 18.2 to 2.9% [8] by the use of the IMA instead of only venous grafts there is a frequent use of arterial grafts nowadays. Weiss at al. presented a wide meta-analysis with n=79,063 patients and concluded that BIMA grafts should be used as gold standard [9]. 

Because of these relevant aspects to use the IMA for grafting, there are a lot of ongoing analyses to find out the predictors of critically reduced sternal blood flow in the use of IMA grafts. The method of intraoperative preparation of the IMA either as pedicle or skeletonized is one of the possible modifications with impact on the SWI [10], [11]. Postoperative wound treatment with negative pressure wound therapy also seems to be effective to optimize the rate of SWI [12]. 

**Figure 3:**
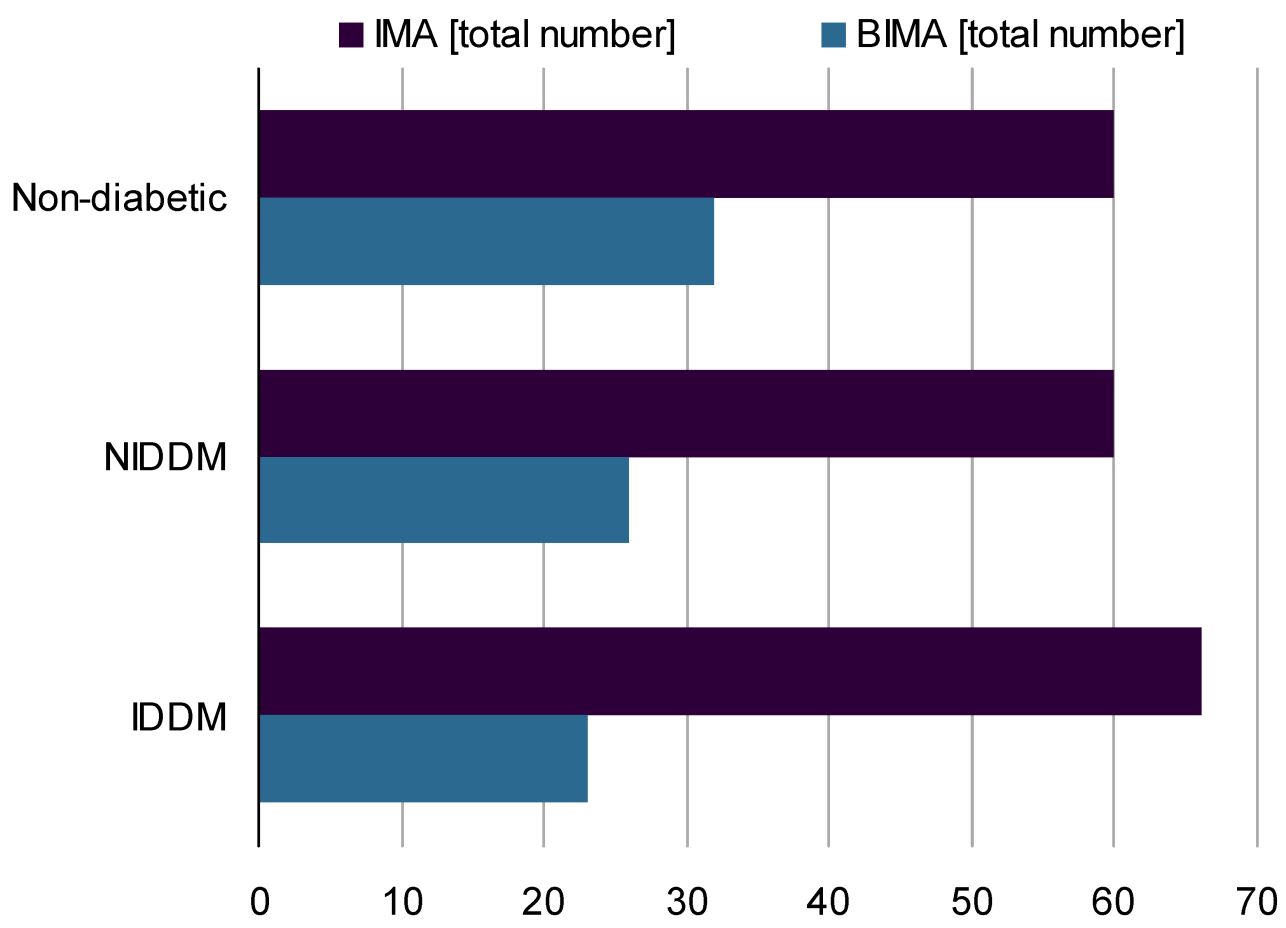
Total numbers of arterial grafts: unilateral internal mammary artery (IMA), bilateral internal mammary artery (BIMA), NIDDM: non-insulin-dependent diabetes mellitus, IDDM: insulin-dependent diabetes mellitus.

In this study, the majority of patients was treated by the use IMA or BIMA grafts (Figure 3 (Fig. 3)). 

In diabetics, the HBA1c is proved as individual predictor for SWI. An increase of more than 9% is highly significantly correlated with postoperative mediastinitis and mortality [13]. The strict perioperative management of blood-glucose level in diabetic patients is proven as an important factor of SWI and the global outcome [14].

This shows the relevance of the diabetes care starting already in the planning of a coronary surgery and in its course during the hospital stay in order to reduce specific risk factors in diabetic patients.

## Conclusion

Diabetic patients suffer from a higher incidence of SWI undergoing coronary artery bypass surgery. The proven risk factors are the pre- and perioperative management of the blood-glucose level. Obesity is still an outstanding factor promoting SWI.

The use of IMA grafts may be an additional risk factor for SWI caused by the reduced blood supply, but the long-term outcome compensates the short-term perioperative complications. The use of BIMA grafts is still in discussion. In our data the use of BIMA was not associated with a significant higher morbidity, the first results of the ART-Trial documents excellent outcome but with a trend towards a higher rate of SWI (0.6% vs 1.9%; [15]). The method of subtle skeletonizing preparation of the IMA grafts preserves the sternal blood supply. Postoperative local treatment of the incision site may reduce the rate of SWI. 

## Notes

### Competing interests

The authors declare that they have no competing interests.

## Figures and Tables

**Table 1:**
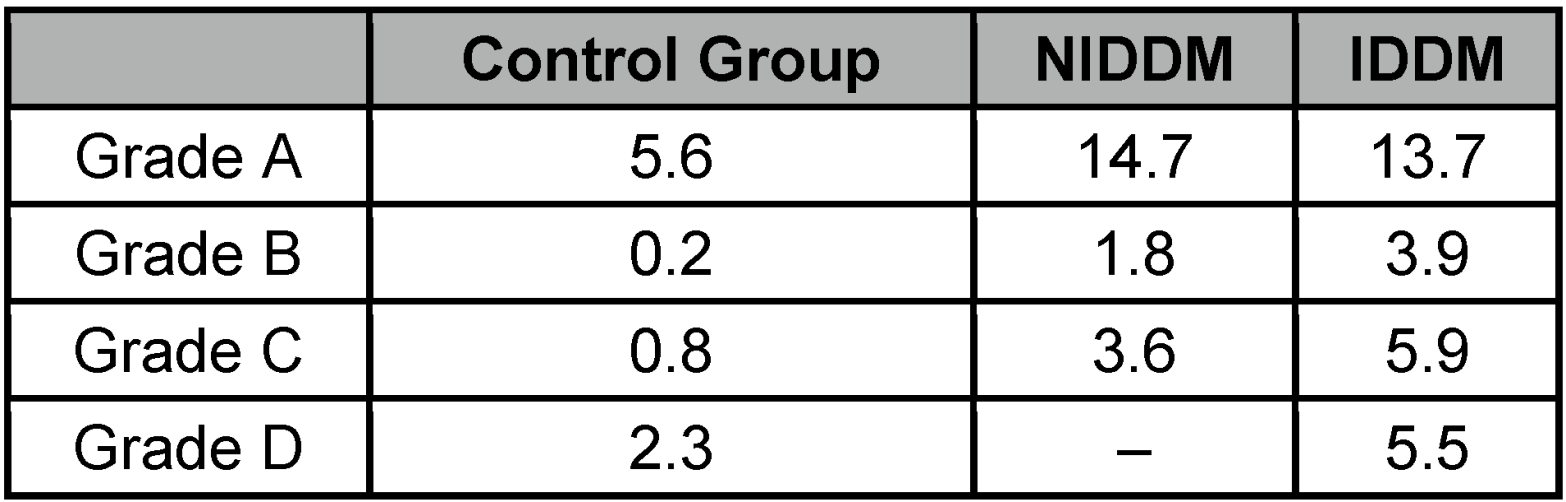
Sternal wound infections after median sternotomy
